# LUCS: a high-resolution nucleic acid sequencing tool for accurate long-read analysis of individual DNA molecules

**DOI:** 10.18632/aging.103171

**Published:** 2020-04-28

**Authors:** Sofia Annis, Zoë Fleischmann, Robert Logan, Zachary Mullin-Bernstein, Melissa Franco, Josefin Saürich, Jonathan L. Tilly, Dori C. Woods, Konstantin Khrapko

**Affiliations:** 1 Department of Biology, Laboratory of Aging and Infertility Research, Northeastern University, Boston, MA 02115, USA; 2 On leave under a Student Exchange Program from the Institut für Pflanzengenetik, Leibniz Universität Hannover, Hannover D-30419, Germany

**Keywords:** DNA, LUCS, sequencing, mutation, chimera, aging, cancer

## Abstract

Nucleic acid sequence analyses are fundamental to all aspects of biological research, spanning aging, mitochondrial DNA (mtDNA) and cancer, as well as microbial and viral evolution. Over the past several years, significant improvements in DNA sequencing, including consensus sequence analysis, have proven invaluable for high-throughput studies. However, all current DNA sequencing platforms have limited utility for studies of complex mixtures or of individual long molecules, the latter of which is crucial to understanding evolution and consequences of single nucleotide variants and their combinations. Here we report a new technology termed LUCS (Long-molecule UMI-driven Consensus Sequencing), in which reads from third-generation sequencing are aggregated by unique molecular identifiers (UMIs) specific for each individual DNA molecule. This enables in-silico reconstruction of highly accurate consensus reads of each DNA molecule independent of other molecules in the sample. Additionally, use of two UMIs enables detection of artificial recombinants (chimeras). As proof of concept, we show that application of LUCS to assessment of mitochondrial genomes in complex mixtures from single cells was associated with an error rate of 1X10^-4^ errors/nucleotide. Thus, LUCS represents a major step forward in DNA sequencing that offers high-throughput capacity and high-accuracy reads in studies of long DNA templates and nucleotide variants in heterogenous samples.

## INTRODUCTION

Every area of biological and biomedical research is rooted one way or another in understanding the precise order of nucleotides in DNA and RNA molecules, and how changes in these sequences subsequently alter downstream function and phenotype within and across generations. These principles apply to all living organisms, as well as to entities such as viruses that are considered by many as non-living organisms. Nucleotide sequence analysis has evolved considerably since Holley and colleagues reported the first complete sequence of a nucleic acid in 1965 [1]. This breakthrough was followed a little over 10 years later with a report from Sanger on the sequence the first DNA genome [2, 3]. These pioneering studies, and those of Maxam and Gilbert [4], provided the early foundation for technology improvements, including the dideoxy chain-termination method or Sanger sequencing [5], which collectively represent what is referred to as first-generation sequence analysis. With the subsequent discovery that the use of radioactive or fluorescent probes to infer nucleotide sequences could be replaced with a luciferase-based pyrophosphate synthesis method referred to as pyrosequencing [6], commercial next-generation sequencing (NGS) was born. Continued improvement in this technology, which relied on specially designed machines capable of performing tremendous numbers of sequencing reactions in parallel, enabled rapid development of high-throughput DNA sequencing that defined the era of second-generation sequencing. Even with these advances, however, all technologies to this point required target DNA amplification. The ability to perform single-molecule sequencing (SMS), and thus minimize biases and errors inherent in DNA amplification, heralded the transition to third-generation sequencing [7, 8].

One of the first widely used third-generation sequencing technologies is the single molecule real-time (SMRT) platform from Pacific Biosciences (PacBio), which offers both high throughput capacity and long reads (10-kb or more). However, a significant limitation to PacBio sequencing is the relatively high first-pass error rate, which is around 8–11%. While the addition of ‘bell adapters’ to sequencing templates allows the templates to be read multiple times in a continuous circle, resulting in a highly accurate circular consensus sequence (CCS), the CCS approach is limited to application with short DNA fragments due to constraints on how long the polymerase remains active. Without an effective way to correct for errors, PacBio cannot be used to accurately sequence long molecules from heterogeneous DNA mixtures. Along with the PacBio SMRT platform, nanopore sequencing, such as that commercialized by Oxford Nanopore Technologies (ONT), represents yet another example of the evolution of DNA sequencing technology [9]. Coupled with considerable improvements in chemistry and software, current versions of ONT sequencing platforms, such as MinION, produce consensus genome assemblies with an error rate less than 1.0% [10]. In parallel to this, unique molecular identifiers (UMI) – random oligonucleotide sequences specific to individual molecules that were first introduced to count molecules in a sample [11], have been employed in error correction approaches [12, 13]. However, high fidelity analysis of single nucleotide variants (SNVs) and their combinations in individual long molecules, especially in heterogenous samples, still remains a significant challenge.

Here we report the development of a new DNA sequencing tool termed Long-molecule UMI-driven Consensus Sequencing or LUCS [14], which we have used with either PacBio or ONT platforms. The LUCS technology utilizes 5' and 3' UMIs incorporated into each individual DNA molecule. permitting the construction of consensus genome sequences from analysis of individual long molecules in complex DNA mixtures. This enables construction of consensus DNA sequences from analysis of individual long molecules, as well as in-silico detection and removal of chimeras. Use of LUCS increased sequencing accuracy of the ONT MinION platform from ~85% to 99.99% (i.e., 1X10^-4^ errors/nucleotide). This vast improvement in accuracy and resolution over current DNA sequencing approaches, together with the inherently high resistance of LUCS to errors introduced through PCR, represents a significant step in the evolution of nucleic acid sequence analysis, with immediate applications to a broad array of critical research topics ranging from aging and cancer to mtDNA inheritance and organismal evolution.

## RESULTS

### Pair-end UMI clustering

**Figure 1:**
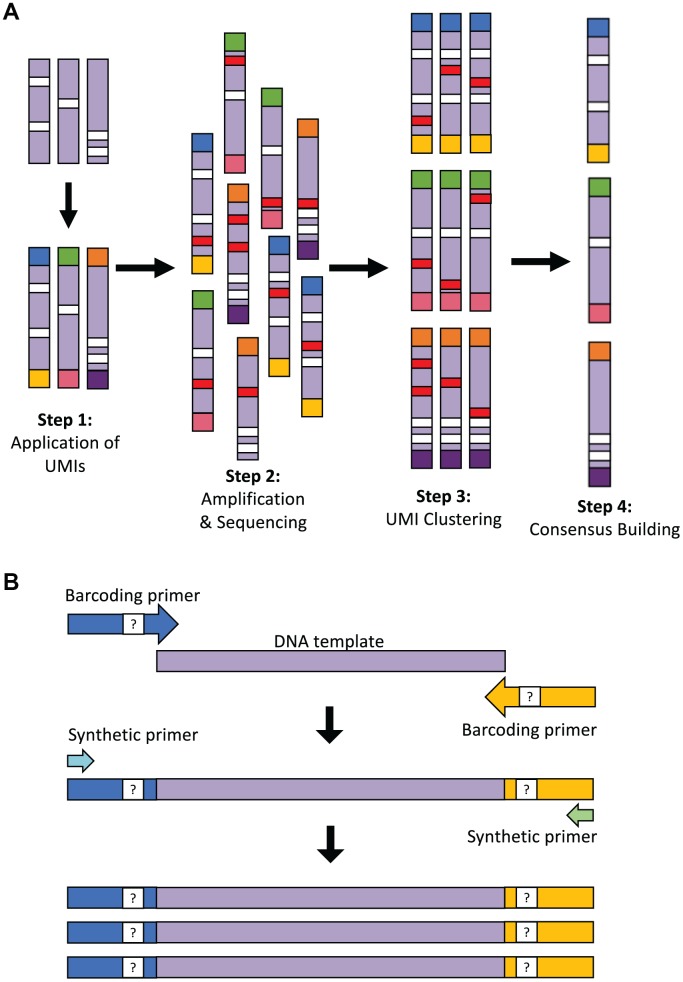
**Overview of the LUCS technology.** (**A**) Each individual DNA molecule in a complex mixture, bearing its own unique pattern of mutations (white), has a UMI applied to it via PCR (each UMI represented by a different end-color), which is specific for that molecule (Step 1). The pool of DNA molecules is then amplified and sequenced (Step 2), during which time artefacts (i.e., PCR errors and sequencing errors) are introduced in a random fashion across molecules (red). All reads are then clustered based on their UMI (Step 3), and a consensus read is built for each molecule (Step 4). This final step removes random errors introduced during the process (red) but retains true mutations (white) found in the original molecule and in all amplicons of that molecule. (**B**) Two-step PCR process for UMI application and dilution. In the first 4 cycles of PCR, the targeted DNA template is amplified by 125-bp oligonucleotide barcoding primers, each containing a random UMI sequence. The initial reaction is then diluted 25-fold within a larger PCR reaction containing only synthetic primers that amplify the UMI-containing molecules after 45 additional cycles of PCR. The resultant elimination of barcoding primer 're-priming' allows for high-resolution pair-end clustering and, in particular, the detection and removal of chimeras (artificial recombinant molecules) caused by PCR jumping.

To perform proof-of-concept testing for LUCS (Figure 1A) on a single-cell analytical level, we used mice with an amino acid substitution (D257A) in the nuclear-encoded DNA *polymerase-γ* (*Polg*) gene [15]. Homozygous *Polg* mice (*Polg*^D257A/D257A^) exhibit an elevated rate of accumulation of mtDNA mutations, reaching ~13.6 mutations per mtDNA molecule during early adulthood, and thus serve as an excellent model for testing the sensitivity of SNV calling in our sequencing strategy. We selected oocytes as a prime single-cell study target since these cells contain an abundance of mitochondrial genomes for analysis. In our initial experimental design, UMIs were applied with barcoding primers and amplified in the same reaction with synthetic primers that capture each UMI sequence (Figure 1A; see also Table 1). Despite a low ratio of barcoding primer (1 μM) to synthetic (10 μM) primer concentration, the barcoding primer was sufficiently active in later stages of PCR to reassign UMIs to molecules that had already received them. This resulted in a complicated pair-end network of clusters of diminishing size. The inability to perform simple pair-end matches between the UMIs made chimera detection and accurate data analysis nearly impossible. Our methodology was therefore modified into a two-step PCR that allows for a 25-fold dilution of the barcoding primers following the four initial cycles when the UMIs are applied (Figure 1B; see Materials and Methods for details). This resulted in a vastly higher resolution of pair-end clustering, which allowed for identification of chimeras. This also supported the accuracy of the clustering algorithm to appropriately identify reads with matching UMIs.

### Consensus sequence analysis

Following UMI-based clustering, a consensus of each cluster was generated. Support for a variant was assessed using reads aligned to this consensus in two ways: base-called support and signal support. Base-called support reports the fraction of aligned reads that support the variant at a given position in the consensus. Meanwhile, signal support refers to the raw feature files (in fast5 format) that estimate the likelihood of the variant. The distinction between base-called and signal support fractions is particularly important here because of the atypical GC-skew of mtDNA. Raw signal support was used to identify spurious variants that could arise from training the neural network of the nanopore base-caller for more standard or methylated genomes. A variant with a high base-called support but low signal support would support that the variant is a false positive, whereas the opposite would support a true variant despite lower support from the read alignment. Training a mtDNA-specific model for base calling is not possible due to the limited training of the ONT base caller, and this would result in over-fitting.

**Figure 2:**
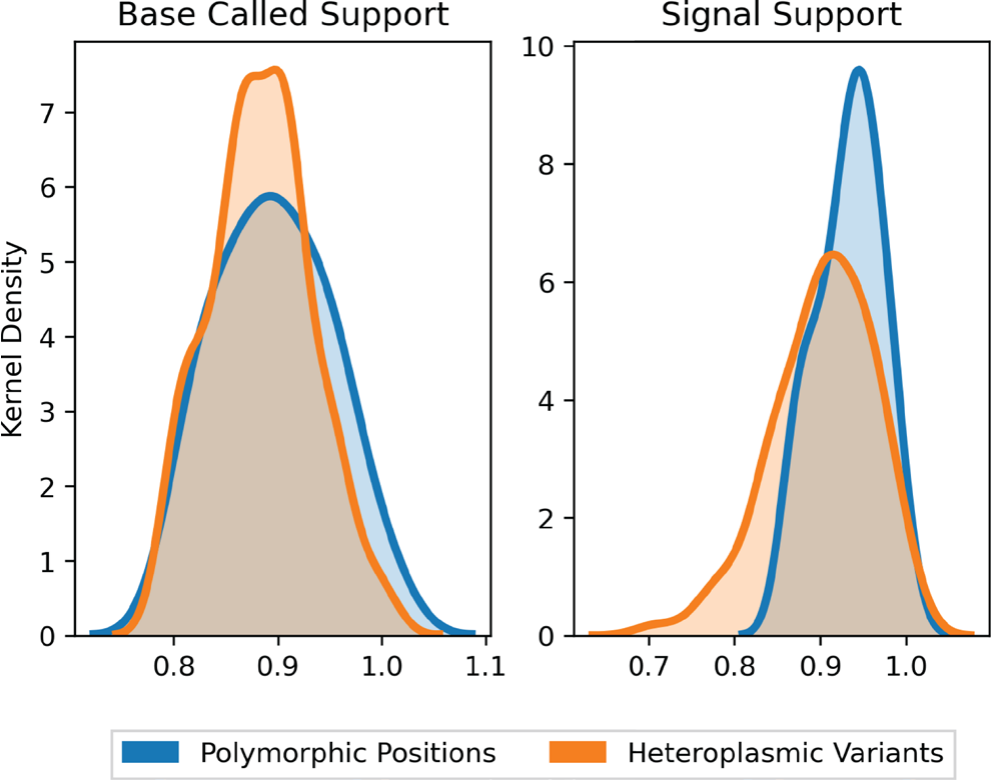
**Support fraction distributions for polymorphic and heteroplasmic variants.** Average base-called support fractions for polymorphic (blue, n = 36) and heteroplasmic (orange, n = 96) variants were 90.1% ± 1.7% and 89.2% ± 0.4%, respectively (mean ± SEM). Likewise, signal support fractions were comparable across polymorphic (93.5% ± 0.7%) and heteroplasmic (92.1% ± 0.6%) variants (mean ± SEM). Distributions are Kernel Density Estimates of base-called and signal support fractions, as determined by nanopolish for all variants. Base-called support and signal support fraction distributions were not significantly different (P = 0.39 and P = 0.17, respectively).

We filtered sites where the base-called support fraction (viz. the percentage of reads within the cluster that contained a given base) for the wild type variant was less than 0.2, yielding a total of 132 putative variants from 12 molecules. Of these, the average base-called support across all variants was 89.4% (with average signal support of 91.7%), and 95.7% of all variants had raw signal support ranging between 80–100% (Figure 2). Positions with signal support lower than 80% could be PCR artefacts from misincorporation of a nucleotide by the Taq polymerase early in the PCR cycling. Five such variants with signal support less than 80% showed support on only one strand and were therefore excluded from further analysis. Variants with low signal support, viz. those below 80%, were randomly distributed across consensus sequences, and no consensus contained more than one variant with low signal support. The presence of variants with high signal support (80% or higher) within the same consensus sequences suggests that the low support variants are a product of random error and not poor-quality clustering or issues with consensus building.

To further corroborate the sensitivity of base-called and signal support for variants, we next compared polymorphic sites to heteroplasmic variants. Polymorphic sites were defined as the variant positions that are found in all molecules sequenced by Sanger from the same sample. Here, "heteroplasmic variants" refers to the frequency at which a variant occurs in the sample, not in the cluster of reads that generate the single molecule consensus. Both heteroplasmic and polymorphic variants would be expected in all reads of the same cluster, but only polymorphic variants would be expected in all reads across all clusters. Therefore, within a cluster of reads that represents a single molecule, base-called support and signal support for both heteroplasmic and polymorphic variants should be similar. The average base-celled support fractions for polymorphic (n = 36) and heteroplasmic (n = 96) variants were comparable at 90.1% ± 1.7% and 89.2% ± 0.4%, respectively (mean ± SEM, P = 0.39). Likewise, average signal support fractions were comparable in heteroplasmic (92.1% ± 0.6%) versus polymorphic (93.5% ± 0.7%) (mean ± SEM, P = 0.17) variants (Figure 2). Collectively, the consistency of support fractions between polymorphic sites and heteroplasmic variants strongly supports that the lower frequency variants are of high quality.

### Verification of LUCS mutation frequency analysis by Sanger sequencing

**Figure 3:**
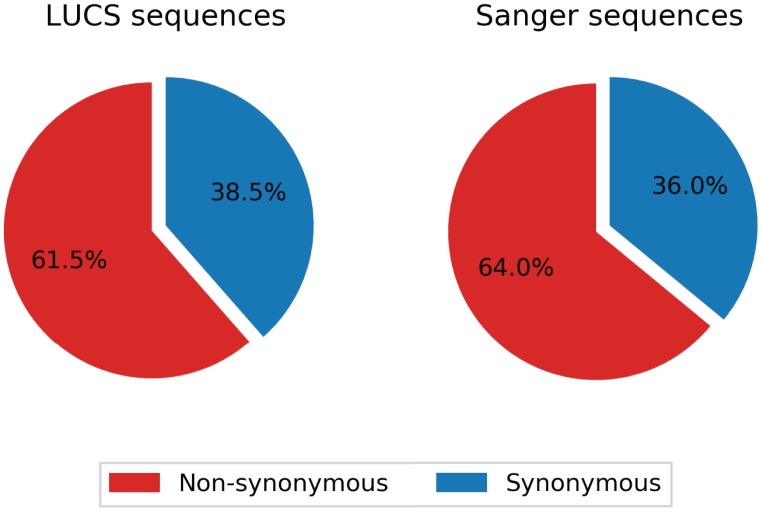
**Comparison of synonymity distributions between the LUCS and Sanger sequencing datasets.** Support fractions above 80% for LUCS and Sanger sequencing methods were comparatively analyzed, and display similar proportional synonymity in coding regions, indicative of low error rate.

**Figure 4:**
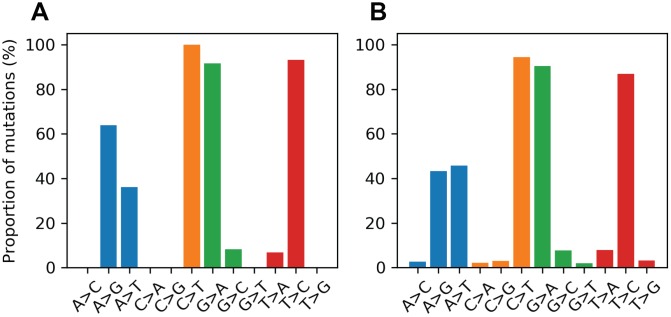
**Proportional mutational spectra for the LUCS and Sanger sequencing datasets.** (**A**, **B**) The mutation spectrum was determined for each reference nucleotide for the LUCS (**A**) and Sanger sequencing (**B**) datasets. Each bar represents the proportion of a variant for a given reference base. For example, the A>G bar is the number of A>G mutations divided by the number of mutated positions that are adenines in the reference sequence. For cytosine, guanine and thymine positions, both LUCS and Sanger mutations exhibited a strong bias towards transitions. Adenine positions were more likely to mutate as a thymine transversion than as a transition in the Sanger dataset, which was reflected to a slightly lesser degree in the LUCS dataset.

**Figure 5:**
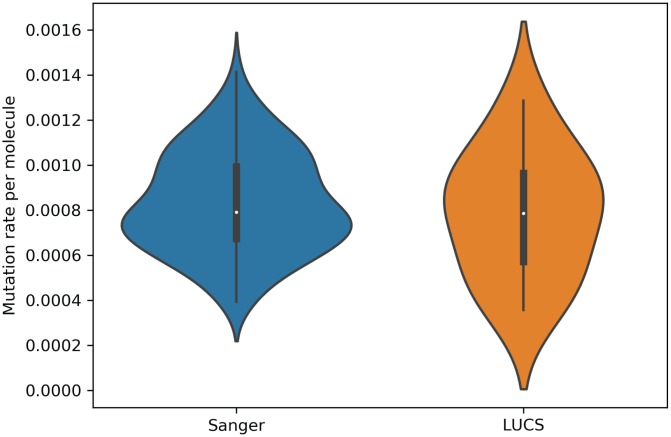
**Mutation rates estimated for single molecules from Sanger sequencing and LUCS datasets.** Violin plots showing that mutation rates per molecule sequenced, determined by dividing the number of mutations by the coverage of a given molecule, were similar between the two technologies (P = 0.12; see text for details).

Mitochondrial DNA from the same oocyte was amplified in single-molecule PCRs without UMI primers and then Sanger sequenced to determine if LUCS variants demonstrated a distribution similar to variants identified by Sanger sequencing. We observed that LUCS variants with support fractions above 80% were 38.5% synonymous, compared to 36.0% using Sanger sequencing (Figure 3). A characteristic feature of Sanger-sequenced variants was a relatively high proportion of transversions, with 45.7% of mutated adenines converted to thymine. While this mutational profile was not observed in variants identified by LUCS (only 36.8% of adenines mutated as transversions), adenine to thymine transversions were more frequently represented versus all other transversions (Figure 4). Finally, the mutation rate associated with variants identified using Sanger sequencing was 8.3X10^-4^ (± 2.11X10^-5^) mutations/bp or ~13.60 mutations per mitochondrial genome. Using LUCS, the mutation rate was 7.9X10^-4^ (± 8.2X10^-5^) mutations/bp or ~12.81 mutations per mitochondrial genome (mean ± SEM) (Figure 5), in close alignment with Sanger sequencing (P = 0.12).

## DISCUSSION

Nucleic acid sequencing has seen tremendous improvements over the last 5 decades, with current third- generation sequencing platforms offering high throughput, single-molecule capacity [16]. However, even the most advanced third generation sequencing technologies (ONT and PacBio) that involve PCR produce sequences with error rates that are not compatible with high-resolution analysis of SNVs in long DNA molecules. Additionally, both ONT and PacBio sequencing rely on massive (~30-fold) consensus reads from multiple different molecules, generating sequence information on average genomes, not single molecules. In other words, individual sequence reads are essentially unrecognizable. The challenge of obtaining high-resolution reads across long spans of DNA are complicated even further when these analyses are performed with complex or heterogenous nucleic acid mixtures.

Consensus sequence building strategies are guided by support fractions. These are the proportion of single reads within a consensus that support the calling of a particular variant, and thus serve as essential indicators of how reliable a given variant is. For example, if the reference base is adenine, and 6% of reads are adenines, 1% are cytosines, 93% are guanines and none are a thymine, the support fraction is 93%. Because inherent error rates in third-generation sequencing fluctuate around 10–15%, support fractions that are significantly lower than 80% raise suspicion over the reliability of an identified variant. Any given consensus sequence has a broad range of support fractions for its identified variants. The implication of this is that low support fractions are not due to widespread quality issues associated with reads in a given consensus or with errors in clustering, but instead arise from more localized problems such as PCR error (e.g., comparable representation of two nucleotides at a given site) or context-specific alignment and base-calling errors (e.g., homopolymer tracts are challenging to base-call, and can also offset alignments if mutations occur within or near such tracts). By setting our consensus support fraction threshold with LUCS to 80%, it is possible that some genuine mutations were lost, but this comes with high confidence in the variants retained. Additionally, intermediate variants can always be processed for manual inspection to make the final call of whether to keep or drop a given variant. For variants identified with an 80% or greater support fraction, it would be nearly impossible for an error at any step in the process to accumulate into such a high support fraction. The only exception to this is first-cycle PCR errors; however, at present there is no PCR-dependent sequencing method that can detect or eliminate this source of potential error.

Molecule-by-molecule sequencing has been performed using the PacBio circular consensus sequencing (CCS) technology, in which a dsDNA molecule capped with two hairpins (‘bell adapters’) is sequenced several times in a rolling or circle fashion to produce several reads of the same molecule. These reads are then combined into a single consensus sequence. The total read length of CCS plateaus around 50-kb. However, to obtain the accuracy needed for high resolution studies of individual DNA molecules, ~10 reads need to be obtained from each molecule. Some scientists have claimed that this limits the length of the original molecule to no more than 5-kb [17, 18]. On the other end of the fragment length spectrum, next generation sequencing (NGS) platforms, such as Illumina, are based on the high-fidelity sequencing of short DNA fragments (<300-bp). The “deep sequencing” or high-coverage version of Illumina can be used to explore microheterogeneity, but this approach yields simply a list of variants and their frequencies. It does not generate reliable information on linkage between variants or “phase”, viz. which variants are positioned on the same DNA molecule.

The development of LUCS offers a solution to all limitations outlined above that exist with current third-generation sequencing platforms dependent on PCR, which we believe will have a major impact on enabling high-resolution analysis of both nuclear and mitochondrial genomes in the context of numerous research directions, including evolution, cancer, aging and non-cancer disease pathogenesis. As an example, we will close with a discussion of why a tool such as LUCS could revolutionize approaches to an area that is of high relevance and priority across the world at the present time. The COVID-19 pandemic has sparked unprecedented efforts to contain the spread of the virus, and to characterize genetic variants of SARS-CoV-2. A global consortium is collecting genetic sequence information on a large scale in an attempt to determine mutational hotspots and the genetic trajectory of the virus. However, sequence data generated thus far that are available in public datasets are limited by incomplete genome coverage and sequencing-associated errors. With policy decisions, diagnostic procedures and treatment protocols rooted heavily in this type of information, the need for high resolution single-molecule analysis, which accurately depicts viral evolution at the level of both individuals and the pandemic, could not be clearer.

To this end, variants of a virus present in an individual are referred to as quasispecies [19, 20]. Identification of quasispecies is considered vitally important for successful development of diagnostic tests, vaccines and drugs, as well as for elucidating complexities in variation, adaptation and infection patterns. The task of exploring quasispecies and the population genomics associated with acute progression of a viral infection in the human body in real time is a daunting one. Ideally, one would like to know the sequences of the entire genomes of a representative subset of the intrapatient viral population, sampled at several time points during the infection. This can be accomplished with great precision by cloning individual viral particles and then sequencing the clones by any convenient sequencing method. This precision, however, comes at a huge financial cost and with very high risks: most notably for the latter, a BSL-3/4 laboratory is needed for handling and cloning of live virus. Additionally, this is a time-consuming process in that, even with a dedicated production line, analytical outcomes are not available for many days.

Together, these issues make current strategies for exploration of viral population genomics within individuals impractical on a large scale; and, they are not rapid enough to adequately address and understand viral evolution throughout the infection process. Accurate analysis of viral variation, and the emergence of quasispecies, during the course of infection may offer new insights into, among other things, the wide-range of patient susceptibility currently observed with SARS-CoV-2, and the role of aging in this, with some patients having no or mild symptoms while others develop severe complications. Indeed, precedent for this exists, with a very recent report of hepatitis-C treatment failure in a subset of patients linked to the emergence of HCV mutations in quasispecies that confer, in those patients, resistance to treatment with conventional therapies [21]. Therefore, detailed sequence information is essential for elaborating the basis of viral adaptive responses in individual hosts during the course of the disease, which will help define the transmission, pathogenicity and treatment strategies for viruses, especially those that are new like SARS-CoV-2, in real time.

It is important to note that a modified CCS strategy was reported recently, with an average accuracy of individual reads of ~0.2%, which increases to ~0.01% if indels in homopolymer tracts are excluded [22]. However, a critical difference between LUCS and this modified CCS approach is that the latter is restricted to analyses of genomic DNA samples that do not require PCR amplification [22, 23]. Hence, this new approach, while accurate, is not feasible with current DNA sequencing platforms that require PCR. This fundamental difference underscores the advantage of using LUCS for high-throughput studies of molecular targets-like mtDNA and viral nucleic acid sequences. In addition to the fact that LUCS can be used with either of the two existing third-generation sequencing platforms (PacBio or ONT), its greatest strength is that LUCS is resistant to the introduction of PCR-based errors. Thus, in sequencing situations where PCR amplification is obligate (e.g., genomic analysis of single cells, or of pathogens in clinical samples where the number of pathogen genomes is limiting), LUCS is superior for achieving the high resolution needed for studies of such complex mixtures.

In closing, LUCS is a tool that dramatically improves the single-molecule sequencing accuracy of whatever base technology it is used with, and its accuracy will only increase as the accuracy of the base sequencing technologies are increased. For example, ONT has recently released a new R10 chemistry, which delivers 95% accuracy in a single read. Testing of LUCS with R10 chemistry is currently underway, and we are confident that our current error rate of 10^-4^ will be improved further. Of final note, long DNA molecules are highly prone to artificial recombination, or PCR jumping, during amplification. If left unaccounted for, PCR jumping fully compromises any attempts to sequence individual long molecules of DNA. The use of two UMIs for each DNA molecule analyzed by LUCS, one at the 5’-end and the other at the 3’-end, enables in-silico detection and removal of chimeras prior to final genome assembly.

## MATERIALS AND METHODS

### Animals and sample collection

All studies with animals reported herein were reviewed and approved by the institutional animal care and use committee of Northeastern University. Heterozygous mice with a single amino acid substitution (D257A) in the nuclear-encoded DNA *polymerase-γ* gene (*Polg*^D257A/+^) were obtained from the Jackson Laboratory (Bar Harbor, ME, USA) and bred to generate homozygous mtDNA mutator mice (*Polg*^D257A/D257A^) [15, 24]. Oocytes were collected after superovulation of young adult (2-month-old) homozygous female mice and denuded of all adherent somatic cells, as detailed previously [24]. Individual oocytes were incubated in 1 μl of lysis buffer (10 mM EDTA, 0.5% SDS, 0.1 mg/ml Proteinase-K) for 3 hours at 37° C and then stored under mineral oil at –80° C.

### Barcoding PCR

For barcoding primers, we used 125-bp oligonucleotides with three distinct regions. The 5’-end was designed as a 64–73-bp synthetic code devoid of guanines, followed by a random 24-bp barcode also devoid of guanines, and ending with a 28–37-bp 3’-end complementary to the target DNA sequence. The synthetic primers were comprised of the first 29-bp of the 5’-end of the corresponding barcoding primer (Table 1). The initial 4 cycles of PCR were conducted in 2-μl reactions containing 1X-concentrated LA Taq reaction buffer (Takara Bio USA, Mountain View, CA, USA), 0.2 mM of each dNTP, 2.5 μM of each barcoding primer, 10 μM of each synthetic primer, and 0.1 units of Hot Start Ex Taq DNA Polymerase (Takara Bio USA), along with lysate prepared from individual oocytes as follows: lysate (1-μl frozen stock; see above) was diluted 10,000-fold in ultrapure water, resulting in an estimated 10 mtDNA molecules per reaction well. Reactions were cycled at 95° C for 30 sec of denaturation followed by 14 min of combined annealing and extension at 68° C. After 4 cycles, reactions were held at 68° C while 48-μl of additional barcoding primer-free PCR mix was added, bringing the final 50-μl reaction to 1X-concentrated LA Taq reaction buffer, 0.2 mM of each dNTP, 0.1 μM of each barcoding primer, 10 μM of each synthetic primer, and 1.25 units of Hot Start Ex Taq DNA Polymerase. Reactions were continued for an additional 45 cycles as described above.

**Table 1:** Sequences of oligonucleotide primers, in 5’ to 3’ orientation, utilized for UMI-based barcoding and PCR amplification (H = A, C or T).

**Primer name**	**Primer sequence**	**Secondary bar-code sequence**	**Primer location**
NPb02H24m3092F (barcoding primer)	CCACTACTCACACACCAATTCCTCTCATTACCACGCACTACCTATTAGATGCTGATGACGCGCTHHHHHHHHHHHHHHHHHHHHHHHHCTCCATTCTATGATCAGGATGAGCCTCAAACTCCAAA	ATGCTGATGACGCGCT	3092 Forward
NPbAdPr (synthetic primer)	CCACTACTCACACACCAATTCCTCTCATTA	N/A	5’-end of NPb02H24m3092F
NPc02H24m786R (barcoding primer)	CCCACACTACAAAACCCACTCATATACACTACACTCTATCAACATACTATCATGCGAGACTATCGCGAHHHHHHHHHHHHHHHHHHHHHHHHGCCCATTTCTTCCCATTTCATTGGCTACACCTT	TGCGAGACTATCGCGA	786 Reverse
NPcAdPr (synthetic primer)	CCCACACTACAAAACCCACTCATATACACT	N/A	5’-end of NPc02H24m786R
3092F	CTCCATTCTATGATCAGGATGAGCCTCAAACTCCAAA	N/A	3092 Forward
3140F	CGGAGCTTTACGAGCCGTAGCCCAAACAAT	N/A	3140 Forward
3003R	GACTTAATGCTAGTGTGAGTGATAGGGTAGGTGCAA	N/A	3003 Reverse
3031R	GGGTGTGGTATTGGTAGGGGAACTCATAGACTTA	N/A	3031 Reverse

Primer location determined from the indicated position in the reference mtDNA sequence (GenBank AY172335.1).

### Library preparation and sequencing

For this experiment, 10 wells of barcoding PCR product were pooled and sequenced. Based on mtDNA copy number estimates from mtDNA content per mouse oocyte [25], this yielded ~100 unique molecules per sample. Products were cleaned using SPRIselect beads (Beckman Coulter Life Sciences, Indianapolis, IN, USA) at 1:4 ratio of product to bead-buffer to diminish the retention of short, non-target by-products. The QuantiFluor dsDNA System (Promega, Madison, WI, USA) was used to quantify DNA concentrations, and 1.5 μg of cleaned DNA was prepared using the 1D amplicon/cDNA by Ligation SQK-LSK-109 protocol (ACDE_9064_v109_revD_23May2018; Oxford Nanopore Technologies, Oxford, United Kingdom) for sequence analysis using a MinION R9 flow cell and MinION software version 19.10.1 (Oxford Nanopore Technologies). Sequence data were base called using Guppy software (version 2.3.7) and the dna_r9.4.1_450bps_flipflop.cfg model (Oxford Nanopore Technologies).

### Single-molecule PCR and Sanger sequencing

Lysate from individual oocytes was serially diluted in order to perform single-molecule PCR, wherein each amplicon originates from a single mtDNA template, as described [26]. In brief, lysate was diluted 300,000-fold in ultrapure water, with approximately 1/3 of the wells being positive and 2/3 being negative for mtDNA. Mitochondrial DNA was initially amplified with primers 3092F and 3031R (Table 1) in 15 μl reactions using Q5 Hot Start Polymerase (New England Biolabs, Ipswich, MA, USA), with final reagent concentrations of 1X-concentrated Q5 reaction buffer, 0.2 mM of each dNTP, 10 μM of each primer and 0.3 units of Q5 Hot Start Polymerase. Reactions were cycled 45 times (30 sec of denaturation at 95° C followed by 16 min of combined annealing and extension at 68° C). Following the initial cycles of PCR, amplicons were re-amplified for 15 additional cycles with Hot Start Ex Taq Polymerase using primers 3140F and 3003R (Table 1) and the following reagent concentrations: 1X-concentrated LA Taq reaction buffer, 0.2 mM of each dNTP, 10 μM of each synthetic primer and 0.15 units of Hot Start Ex Taq DNA Polymerase. Amplicons were sequenced across 24 sequencing reactions on a 3720xl DNA Analyzer (Applied Biosystems, Foster City, CA, USA). Reads were assembled and aligned against the C57BL/6 mouse mtDNA reference genome (GenBank AY172335.1). CodonCode Aligner software (CodonCode Corporation, Centerville, MA, USA) was used for assemblies and alignments, and each mutation identified was manually confirmed. Sequences with overlapping peaks were discarded as mixed molecules derived from multiple, rather than single, templates.

### Data processing and analysis

Short reads were removed with Filtlong (https://github.com/rrwick/Filtlong) to a minimum size of 13.7-kb, which is 300-bp shorter than expected length of a UMI-labelled PCR fragment. Reads were then processed in Porechop (https://github.com/rrwick/Porechop) to remove residual ONT adapters. Forward and reverse reads were sorted using Cutadapt (http://journal.embnet.org/index.php/embnetjournal/article/view/200) in paired-end mode. The reverse complement of the reverse reads (https://github.com/lh3/seqtk) and the forward reads were concatenated into a single FASTQ file. Read UMIs were extracted using the template sequence in Cutadapt, leaving two FASTA files: forward-read UMIs and reverse-read UMIs. Read UMIs were clustered in python using a network-based approach, which leverages the repetitiveness of read UMIs and the linkage information between forward- and reverse-read UMIs. Chimeric clusters were pruned by removing read UMIs if metric longest common subsequence (LCS) exceeded 0.125 from the largest UMI in the cluster. This limit was chosen because it allows for no more three differences between read and centroid, in line with the expected error rate of ONT-based reads. Metric longest common subsequence is defined for two sequences, a and b, of length |a| and |b|, where:

$$metric\;LCS=\frac{\left|LCS(a,b)\right|}{\max(|a|,\;|b|)}$$

Filtered clusters were written to separate FASTQ files. Reads were aligned to the mtDNA reference sequence (GenBank AY172335.1) using minimap2 (https://github.com/lh3/minimap2) and were polished with medaka (https://github.com/nanoporetech/medaka). Genotypes were called using nanopolish (https://github.com/jts/nanopolish) on all medaka alignments to confirm that no chimeras were present, and to generate base-called and raw signal support fractions for all variants. Compiled data (mean ± SEM) were analyzed by ANOVA and Student’s *t*-test.

For this study, a total of 548,000 reads were sequenced, of which 78,105 (14.25%) met the hard length threshold of 13,700-bp. Forward and reverse reads sorted in Cutadapt yielded 31,367 forward reads and 15,036 reverse reads that had adapters on both ends, for a total of 46,403 reads remaining at this stage. Only 11,588 reads had UMIs at both ends that met the length and quality cut off for UMIs. Of these UMIs, 1,001 5'-UMIs and 892 3'-UMIs repeated more than once in the dataset, which accounted for 5,208 and 5,732 reads averaging 5.2 and 6.4 repetitions, respectively. In clustering, 1,147 reads were clustered by their 5'-UMIs and 1,645 reads were clustered by their 3'-UMIs. After filtering for chimeras, 12 clusters remained at a minimum depth of 20 reads, with an average depth of 42.1 reads/consensus.
